# Exploring the Effect of Iron Metal-Organic Framework Particles in Polylactic Acid Membranes for the Azeotropic Separation of Organic/Organic Mixtures by Pervaporation

**DOI:** 10.3390/membranes11010065

**Published:** 2021-01-18

**Authors:** Asma Msahel, Francesco Galiano, Martina Pilloni, Francesca Russo, Amor Hafiane, Roberto Castro-Muñoz, Vijay Bhooshan Kumar, Aharon Gedanken, Guido Ennas, Ze’ev Porat, Alessandra Scano, Sofiane Ben Hamouda, Alberto Figoli

**Affiliations:** 1 Laboratory of Water Membrane and Environmental Biotechnology (LMBE), CERTE BP 273, 8020 Soliman, Tunisia; asmamsehel@gmail.com (A.M.); amor.hafiane@certe.rnrt.tn (A.H.); sofianebenhamouda819@gmail.com (S.B.H.); 2 Department of Chemistry, University of Tunis El-Manar, Farhat Hached University Campus, BP n° 94 Rommana, 1068 Tunis, Tunisia; 3 Institute on Membrane Technology, ITM-CNR, Via P. Bucci 17/c, 87036 Arcavacata di Rende (CS), Italy; f.russo@itm.cnr.it (F.R.); a.figoli@itm.cnr.it (A.F.); 4 Chemical and Geological Science Department, Unità di Ricerca del Consorzio Nazionale di Scienze e Tecnologie dei Materiali (INSTM), University of Cagliari, SS 554 Bivio Sestu, 09042 Monserrato (CA), Italy; ennas@unica.it (G.E.); alescano80@tiscali.it (A.S.); 5 Tecnologico de Monterrey, Campus Toluca, Avenida Eduardo Monroy Cárdenas 2000, San Antonio Buenavista, Toluca de Lerdo 50110, Mexico; food.biotechnology88@gmail.com; 6 Department of Chemistry, Bar-Ilan University, Ramat-Gan 5290002, Israel; vijaybhushan86@gmail.com (V.B.K.); Aharon.Gedanken@biu.ac.il (A.G.); 7 Division of Chemistry, Nuclear Research Center-Negev, P.O. Box 9001, Be’er-Sheva 8419001, Israel; poratze@post.bgu.ac.il; 8 Unit of Environmental Engineering, Ben-Gurion University of the Negev, Be’er-Sheva 8410501, Israel; 9 NANOMISENE Laboratory, LR16CRMN01, Centre for Research on Microelectronics and Nanotechnology (CRMN) of Technopole of Sousse B. P334, 4054 Sahloul Sousse, Tunisia

**Keywords:** pervaporation, polylactic acid (PLA), iron metal organic-framework, MOF, methanol, methyl *tert*-butyl ether (MTBE), organic-organic separation

## Abstract

A microporous carboxylate metal-organic framework MIL-100 Fe was prepared as submicron particles by microwave-assisted hydrothermal synthesis (Fe-MOF-MW). This product was explored, for the first time, for the preparation of polylactic acid (PLA) mixed matrix membranes. The produced MOF was characterised by powder X-ray diffraction (PXRD), environmental scanning electron microscopy (ESEM) as well as by thermogravimetric analysis (TGA) and nitrogen adsorption/desorption. The effect of different Fe-MOF-MW concentrations (0.1 and 0.5 wt%) on the membrane properties and performance were evaluated. These membranes were used in the pervaporation process for the separation of methanol/methyl *tert*-butyl-ether mixtures at the azeotropic point. The influence of the feed temperature and vacuum pressure on the membrane performance was evaluated and the results were compared with PLA pristine membranes. Moreover, the produced membranes have been characterised in terms of morphology, MOF dispersion in the polymeric membrane matrix, wettability, thickness, mechanical resistance and swelling propensity. The presence of Fe-MOF-MW was found to have a beneficial effect in improving the selectivity of mixed matrix membranes towards methanol at both concentrations. The highest selectivity was obtained for the PLA membranes embedded with 0.5 wt% of Fe-MOF-MW and tested at the temperature of 25 °C and vacuum pressure of 0.09 mbar.

## 1. Introduction

Pervaporation (PV) is a well-known membrane separation technique, which combines permeation and evaporation processes [1]. PV operates by means of non-porous membranes where a liquid feed is brought in contact with one side of the membrane, while the opposite side is in contact with the permeate in a vapour form. Among the various types of PV applications, the separation of organic-organic mixtures is one of the most studied ones due to its relevant importance in the petrochemical and chemical industry.

PV is particularly recognized with respect to other conventional separation technologies such as chromatography, distillation and extraction, because it often represents the simplest and cheapest way to perform the separation owing to a series of benefits such as the low energy requirements, the high efficiency and the possibility to work without the use of entrainers [1,2].

Nowadays, methanol (MeOH)/methyl *tert*-butyl ether (MTBE) is one of the most studied organic/organic mixtures. MTBE is employed as an octane enhancer of gasoline [1] and it is synthesized by the reaction of isobutylene and MeOH [2]. However, the excess of unreacted MeOH, in the end of the synthesis, must be removed in order to obtain pure MTBE.

It has been reported that certain countries, including the USA, have banned the production and use of MTBE due to its toxicity and the possibility that it can reach and contaminate groundwaters. However, other European and Asian countries are still using this chemical for fuel production [2]. In this regard, mixture of MeOH and MTBE, form an azeotrope at a specific concentration (14.3 and 85.7 wt%, respectively), in which MTBE purification may demand more than two distillation columns, being responsible for the highest energy consumption of the overall production process. Therefore, a more efficient process is required, such as PV. To date, several authors have proposed different types of polymeric and inorganic membranes for such a separation by means of PV, including commercial polyimides [1], polyether ether ketone (PEEKWC) [3], zeolites [4] and polylactic acid (PLA) [5], just to mention a few. A current trend, which is becoming more and more appreciated, lies in the combination of organic polymers with inorganic materials for the fabrication of membranes with superior performance. The so-called mixed matrix membranes (MMMs) combine the advantages of polymer membranes (such as easy preparation procedure, flexibility, low cost) with the benefits stemming from inorganic fillers (e.g., improvement of membrane selectivity and mechanical properties) [6,7].

Recently, we reported on the efficiency of PLA for the azeotropic separation of MeOH-MTBE mixtures [5]. The choice of PLA as a polymer stems from the necessity of finding more sustainable and bio-based materials in the light of reducing the environmental impact of fossil-based polymers which are commonly employed in membrane fabrication [8,9,10]. PLA is an aliphatic thermoplastic polyester. It is derived from lactic acid (obtained from the fermentation of C_6_-sugars) converted into dilactide after self-esterification, which, in turn, is converted into PLA via polymerization. PLA is a compostable, renewable, biodegradable and versatile bio-polymer which is readily processable by the polymer industry [11].

In the current work, a step towards improving the PLA membrane separation properties is proposed, by incorporating into the polymer matrix a metal-organic framework (MOF).

Remarkable results were obtained in PV process throughout the use hybrid membranes fabricated with different MOFs [12,13,14,15,16,17,18,19]. The MOFs’ distinctive characteristics, such high surface area and porosity, low density, adaptable surface property, tuneable pore structure, and rich chemical functionalities are indubitable advantages of membrane separation [20] and represent the opportunities to solve the permeability/selectivity trade-off problem [21]. As a constituent of the membrane, the MOF offers a large surface area for sorption and a mass transfer contribution to the flow through the pores [12]. While rigid frameworks with small pores showed a molecular sieving effect-type [22], flexible structures do not display a clear cut-off effect [23] but exhibited diffusivity also to larger molecules than their pores, with a probable guest inclusion effect [24]. Speculatively, the introduction of MOFs into the MMMs also improves the affinity to some organic compounds over others, due to aromatic entities (generally represented by the linker), polar groups and unsaturated metals that form the framework [25]. For example, in the separation of MeOH/MTBE mixture, the incorporation of the carboxyl-bipyridine [Cu_2_(bdc)_2_(bpy)]_n_ MOF into polyarylethersulfone (PPSU) matrix increased the sorption selectivity and the diffusion selectivity due to the preferential adsorption of MeOH by the MOF [26].

With attention to the separation of MeOH/MTBE mixtures, this research presents for the first time, the preparation of MMMs based on PLA as a polymer and MOF MIL100-Fe [27] as a filler.

This MOF that chemically is an iron (III) trimesate exhibits a microporous architecture (with microporous windows of 5.5 and 8.6 Å) combined with excellent chemical stability and a proved stability in the air [28], water [28] and organic solvents [29]. These characteristics make this structure high manageable and apt to be used as a filler in membrane separation. It is expected iron-based MOF to be a good filler for the PLA membrane, and to improve the selectivity of the membrane in the separation of MeOH/MTBE mixture.

Horcajada’s research group obtained the MIL 100 (Fe) structure in an autoclave starting from iron in a highly acidic medium due to HF and HNO_3_ [27]. MOF has a 3D cubic structure made from oxo-centered trimers of iron (III) octahedra (μ3-O) and trimesate ligands. Each trimer is coordinated to six carboxylates, two coordinated water molecules and F anion. However, the same crystalline structure was obtained in other synthesis conditions, starting from iron salts and in the absence of hydrofluoric acid [30,31].

Here the MIL-100 (Fe) for the incorporation into the PLA matrix is obtained with a green chemistry approach [32] by microwave (MW) irradiation in a domestic microwave oven. We started from water solutions containing the iron nitrate and the trimesic acid without toxic additives (i.e., HF) that also represents a limit for the scaling up [33]. We selected the nitrate salt because it was already preferred over the chloride in the hydrothermal synthesis [31] and gave successfully results in other alternative methods [34], but, as far as we know, it has never been used before under MW for obtaining this iron (III) trimesate structure.

The microwave-assisted hydrothermal method is a convenient synthetic way already used for the MOF synthesis [35]. It is a low cost and rapid method [36,37] compared to traditional autoclave, and it also offers control over the shape and size distribution of particles [38]. The MW method allows obtaining particles with a narrower size distribution, which is particularly desirable in achieving a homogenous dispersion of particles into the polymer matrix [39] during the membrane preparation. In this work, the MW is used as sustainable synthetic method to obtain the MIL-100 (Fe) in water in a short time in line with the green chemistry approaches.

The paper present at first the synthesis of MOF particles in MW and, then, their characterization with different techniques: powder X-ray diffraction (PXRD)), environmental scanning electron microscope (ESEM), thermogravimetric analysis (TGA) and nitrogen sorption at 77 K.

As the second part, the paper presents the preparation of PLA membrane with the MOF as filler. MOF particles (with different loadings) were dispersed in chloroform and PLA was added to fabricate the hybrid membranes by evaporation technique. The resulting membranes were then characterized in terms of swelling degree, visual observations by SEM and mechanical properties. Finally, they were tested for PV separation of MeOH/MTBE azeotropic mixture at different operating temperatures and vacuum permeate pressures. The results were compared with those obtained with pristine PLA membranes.

## 2. Materials and Methods

### 2.1. Materials

PLA was purchased from Cargill-Dow Inc. (Minnetonka, MN, USA) with the trade name of Nature Green 2100D (D % comonomer of up to 1.47 ± 0.2%; highly crystalline). Chloroform stabilized by ethanol was supplied by Panreac (Milan, Italy) with minimum assay >99.0%. MeOH was obtained from VWR chemicals (Milan, Italy) and MTBE 99.9% was purchased from Sigma Aldrich (now Merck, Milan, Italy). For the synthesis of iron-based MOF, iron (III) nitrate nonahydrate (Fe(NO_3_)_3_ × 9H_2_O, 98%), 1,3,5 benzene tricarboxylic acid (H_3_BTC, 95%), ethanol (96%) were purchased from Sigma Aldrich (Merck KGaA, Darmstadt, Germany) and used as received without further purification.

### 2.2. Fe-MOF-MW Synthesis

The MOF particles were prepared via hydrothermal synthesis promoted by microwave irradiation using 1.22 g of iron nitrate and 0.42 g of H_3_BTC. Both reactants were initially mixed under magnetic stirring with 30 mL of bi-distilled water in a 50 mL glass flask. The flask was positioned into a domestic microwave oven operated at 2.45 GHz with an output of 1100 W and connected with a reflux system. The irradiation was carried out for 20 min at 70% power, obtaining a dark orange suspension. After cooling to room temperature, a precipitate was collected by ultracentrifugation at 12,000 rpm for 30 min. The solid was repeatedly washed with bi-distilled water through suspension/centrifugation cycles and finally with ethanol. Thereafter, it was dried out overnight in a glove box. The dry solid (Fe-MOF-MW), which resulted in a soft light orange powder, was further characterised and utilized as filler in the membrane preparation.

### 2.3. Fe-MOF-MW Characterization

The powder X-ray diffraction (PXRD) analyses of Fe-MOF-MW were collected using an AXS Advance powder X-ray diffractometer (Bruker, Billerica, MA, USA, Cu Kα radiation; λ = 0.154178 nm) purchased from Bruker (Billerica, MA, USA) operating at 40 kV/30 mA with a 0.02 step size in the range of 5–40° (2θ).

Thermal behaviour of the sample was examined by thermogravimetric analysis (TGA) using a TG apparatus (series 7) purchased from Perkin-Elmer (Wellesley, MA, USA), under an argon flux and with a heating rate of 10 °C min^−1^. N_2_ physisorption isotherms at −196 °C (77 K) and a P/P_0_ range of 0–1 bar was used to evaluate the porosity in ASAP 2020 Accelerated Surface Area and Porosimetry system purchased from Micromeritics (Norcross, GA, USA). The Surface area was calculated as P/P_0_ = 0.25, the total pore volume was calculated using the single point adsorption at 0.99 P/P_0_. The Hovarth-Kavazoe equation was applied to calculate the total micropore volume and the pore size distribution (PSD). The imaging and morphology were obtained using by FEI QUANTA (Hillsboro, OR, USA).

### 2.4. Mixed Matrix Membrane Preparation

The pristine PLA membrane (M1) was prepared by adding the polymer (10 wt%) to chloroform and stirring the solution at room temperature until complete PLA dissolution (about 5 h). In the case of MMMs, two different Fe-MOF-MW loadings were considered (0.1 and 0.5 wt% for M2 and M3 membrane, respectively). In this case, the proper amount of filler was preliminarily dispersed in chloroform by alternating vigorous magnetic stirring to probe-type sonication (3 cycles of 30 min each one). A homogenous stable mixture with a good filler dispersion was, thus, obtained. PLA (10 wt%) was later added to the mixture and the solution was stirred until complete dissolution of the polymer.

All the membranes were prepared by casting the polymer solution using a manual casting knife with a set thickness of 350 μm. Membranes were formed by solvent evaporation and they were later peeled-off from the glass plate by immersion in water. They were finally dried in the oven at 30 °C for 48 h until use.

### 2.5. Membrane Characterization

#### 2.5.1. SEM Analysis

The morphology of prepared membranes and the distribution of Fe-MOF-MW were investigated by scanning electron microscopy (SEM) and using also a backscattered electron detector (BSD) (Zeiss EVO, MA100, Assing, Milan, Italy). The samples were sputter-coated with gold (sputter machine Quorum Q 150R S, Laughton, UK) before SEM observation. Energy dispersive x-ray analysis (EDX) was carried out by means of Phenom Prox instrument (ThermoFischer Scientific, Rodano (MI), Italy).

#### 2.5.2. Thickness and Contact Angle Measurements

The thickness was measured using a digital micrometer (40E, Mahr, Esslingen, Germany) with an accuracy of ±4 μm. The contact angle measurements were performed using an optical instrument (Nordtestsrl, G-I, Serravalle Scrivia (AL), Italy) by sessile drop method.

#### 2.5.3. Mechanical Properties

Mechanical tests were carried out using a Zwick/Roell universal testing machine, single-column model Z2.5, equipped with a 50 N maximum load cell (BTCFR2.5TN-D09, Zwick/Roell, Ulm, Germany). Membrane samples with a dimension 1 × 6 cm^2^ were used.

#### 2.5.4. Swelling Experiments

For measuring the swelling properties of the membranes, different MeOH:MTBE ratios (0:100; 5:95; 10:90; 14.3:85.7; 25:75; 50:50; 0:100) were used. For each membrane formulation, three small pieces with different shapes were dried, weighed with a digital balance (Gibertini, Crystal 500, Novate Milanese, Italy) before (*W_d_*) and after immersed in a MeOH/MTBE mixture at room temperature for 24 h to reach swelling equilibrium (*W_s_*). After this time, they were taken out and quickly dried with tissue papers by removing the excess liquid from their surface and reweighed. The degree of swelling (*DS*) for each membrane was calculated as follows:

(1) $$DS\left(\%\right)=\frac{W_{s}-W_{d}}{W_{d}}\times 100$$

where *W_s_* and *W_d_* are the weights of the wet and dry membrane, respectively.

#### 2.5.5. PV Tests

PV tests were carried out using a set-up schematically represented and described elsewhere [40]. In brief, a double-jacket reservoir, with a capacity of 300 mL, was filled with the MeOH/MTBE azeotropic mixture (14.3%/85.7%) that was put in contact with a membrane area of 9.6 cm^2^. A digital circulating bath (Thermo Electro Corporation, HAAKE P5, Thermo Fisher Scientific, Rodano, Italy) was used to maintain the feed solution at a specific temperature (25, 35 or 45 °C). The vacuum on the permeate side was varied (0.09; 0.64 and 7.45 mbar) by means of a vacuum pump (Edwards XDS 5, Cinquepascal, Trezzano sul Naviglio Milano, Italy) and controlled by a digital vacuum meter (5Pascal, A921, Trezzano sul Naviglio Milano, Italy). The vapor permeate was collected, during the 5 h of experiment, in a cold trap immersed in liquid nitrogen and analysed with an Abbe 60 type refractometer (60/DR, Bellingham + Stanley Ltd., UK) at 25 °C. After measuring the collected permeate sample, the total flux (*J*), partial fluxes (*J_i_*) and separation factor (α) were determined according to the Equations (2)–(4), respectively:

(2) $$J=\frac{Q}{\mathrm{At}}$$

where Q is the weight of the permeate (kg) collected during specific time t (h) and A is the active membrane area (m^2^):

(3) $$Ji=P_{i}\cdot J$$

where *Ji* is the partial flux of the permeating component *i* in the total permeate flux (*J*) and *P_i_* represents its weight fraction:

(4) $$\alpha =\frac{y_{MeOH}/y_{MTBE}}{x_{MeOH}/x_{MTBE}}$$

where *y* and *x* are the weight fractions of the components in the permeate and feed sides, respectively.

The temperature dependence of the PV flux was studied according to Arrhenius relationships:

(5) $$J=J_{0}\left(-\frac{E_{p}}{R\;T}\right)$$

where *J*_0_ and *E_p_* are the pre-exponential factor and the apparent activation energy, respectively; *R* is the gas constant, and *T* is the absolute temperature.

## 3. Results and Discussion

### 3.1. MOF Characterization

**Figure 1:**
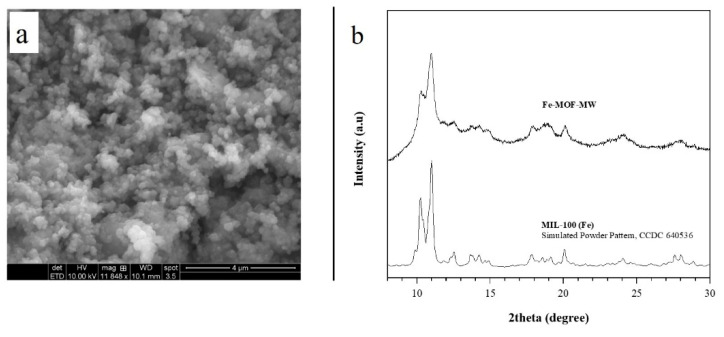
(**a**) ESEM image of Fe-MOF-MW sample; (**b**) experimental PXRD pattern of Fe-MOF-MW sample obtained in this work after 20 min and simulated powder pattern of reference structure MIL-100(Fe) [27].

The ESEM observations reveal the Fe-MOF-MW as a homogeneous sample; it appears in the form of aggregates of spherical particles with an estimated size of 0.1 μm (Figure 1a). The homogeneous morphology and the uniform distribution of particles’ sizes can be attributed to the use of the microwave route [41,42]. It was reported in the literature that for nanoporous materials, the distribution of the particles’ sizes is narrow when microwave energy is employed for the synthesis [43]. One explanation is that microwaves promote more uniform processes during the formation of the nanoscale material. Specifically, some authors explain the narrowed distribution by the faster nucleation of the initial crystallites. Other authors attribute it to a more uniform growth process under microwave irradiation [43].

PXRD analysis reveals that the product of the synthesis has a single crystalline phase; the positions of the peaks between 8° and 30° 2theta (Figure 1b) are consistent with the phase MIL-100(Fe) reported by previous studies [34] and by Garcia Marquez et al. [42] in similar conditions but starting from the iron chloride salt. To the best of our knowledge, so far there are no other results on nitrate use to obtain this MOF in MW.

For clarity, the Figure 1b depicts the simulated powder pattern of MIL-100(Fe) obtained from the structure parameters (CCDC 640,536 from [27]) throughout the MERCURY software [44] provided by the Cambridge Crystallographic Data Centre.

This result confirms the microwave as a quick and valid route to obtain MOFs structures as already described by the literature [45]. Under microwave conditions, the fast heating and the creation of hot spots justify the synthesis rate increase due to the rapid nucleation [46]. Here the MIL-100 type structure is obtained in water within 20 min, unlike the 12 h (or days) required in autoclave or reflux [30,31,47,48].

In Figure 1b a slightly low crystallinity is observed for the Fe-MOF-MW. The literature previously documented the possible presence of an amorphous phase for the MIL-100 structures obtained in water without HF [31]. Such is known as HF, which acts as coordinator modulator [49] improves the crystallinity of product [50]. However, due to the toxicity, the replacement of HF in the synthesis is auspicial, although it means to obtain a product with lower crystallinity [33].

Also, the synthetic method could determine the formation of the amorphous phase. To simplify this interpretation, we consider the nucleation and the crystal growth stage. As mentioned, the microwave irradiation produces fast heating and hot spots, and they induce an acceleration of nucleation rate larger than that in the crystal growth stage [51]. We can speculate that not all the nuclei have the same subsequent growth stage; the nuclei involved in a growth-limited process are not in the conditions to remarkably growing as crystalline solid but remain as amorphous condensed metal-organic species.

**Figure 2:**
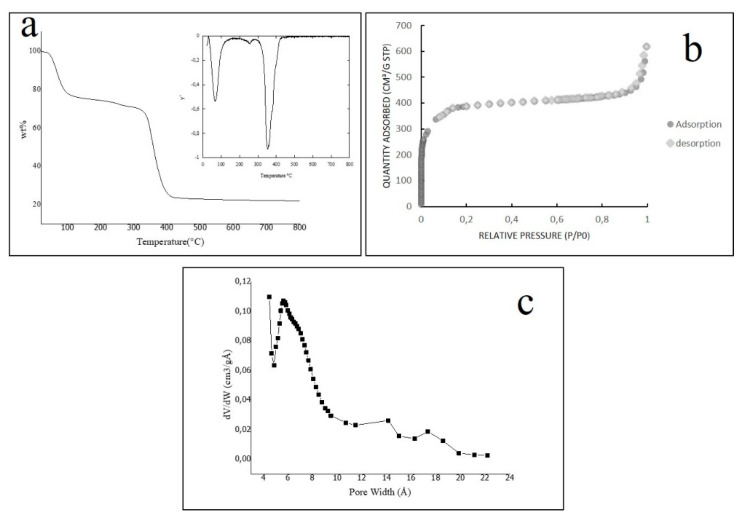
(**a**) TGA curve and TGA derivate for the sample f Fe-MOF-MW (**b**) N_2_ adsorption/desorption isotherm of Fe-MOF-MW (**c**) micropore size distribution calculated in the range 0.01 < P/P_0_ < 0.18 by Hovarth-Kavazoe equation.

The thermogravimetric analysis conducted under an argon atmosphere is depicted in Figure 2a, together with its derivate. A first weight loss of almost 25% was observed between 20 and 150 °C and can be related to adsorbed species’ departure (i.e., water and ethanol molecules). Then, with the increase of temperature, the sample was moderately losing weight, and this was related to the removal of water molecules coordinated with the trimers [27]. The main step of weight-loss is observed between 280 to 430 °C, which is associated with the framework collapse. The complete decomposition of the metal-organic structure gave the oxide Fe_2_O_3_ as the final product at 800 °C. The TGA results agreed with those obtained by Garcia Marquez et al. [40] and those observed for fluorinated structures obtained throughout different synthetic ways [30].

The TGA disclosed two essential aspects of Fe-MOF-MW sample: (a) it is thermostable until 280 °C; (b) it can adsorb species for more than 20% of its weight, due to its porous structure. The nitrogen adsorption/desorption analysis reveals such permanent porosity. The isotherm, shown in Figure 2b demonstrates that the sample includes a large volume adsorbed at low-pressure and no hysteresis; these features are ascribable to a typical type I behaviour [52], which indicates that the microporosity of Fe-MOF-MW is analogous to the structure (obtained by the solvothermal synthesis by Horcajada et al. [27]).

The calculated single-point surface area is 1280 m^2^/g, and the total pore volume is 0.96 cm^3^/g. The values of N_2_ absorption observed in Figure 2b are following those obtained for the F-free structure in water [42], but lower than the values found for the structures obtained in strongly acidic media with HF [53]. It can be explained with a lower surface area of Fe-MOF-MW sample due to a slightly lower crystallinity as discussed before for the PXRD pattern.

The total microporous volume was found to be 0.60 cm^3^/g in the range of 0.01 < P/P_0_ < 0.18 that represents the relative pressures at which all the micropore were filled. In the same range, the pore size distribution (PSD) was calculated as the linear function *dV/dW* (where *V* is the amount of adsorbed nitrogen in the selected range and *W* is the pore size) and depicted in Figure 2c. The distribution curve shows a maximum centered on 5.5 Å indicating a main population of micropores with that size. According to the literature, this MOF structure has microporous windows of 8.6 Å and of 5.5 Å [27], however it is not possible to discriminate those experimentally with the N_2_ probe.

### 3.2. Membrane Characterization

**Figure 3:**
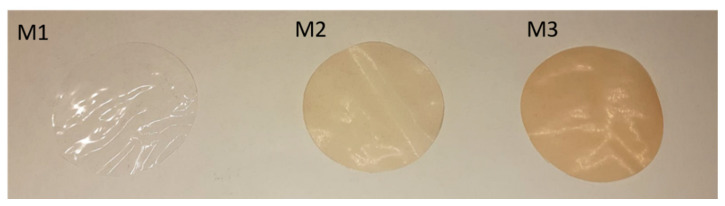
Pictures of M1, M2 and M3 membranes.

Figure 3 shows the picture of prepared membranes. As it is possible to observe, the presence of the filler and its good distribution in the polymer matrix is clearly visible. The transparency of the pristine PLA membrane (M1), in fact, turned to red as the Fe-MOF-MW filler was added into the membranes (M2 and M3). The red colour is due to the presence of iron in the composition of the selected MOF.

**Figure 4:**
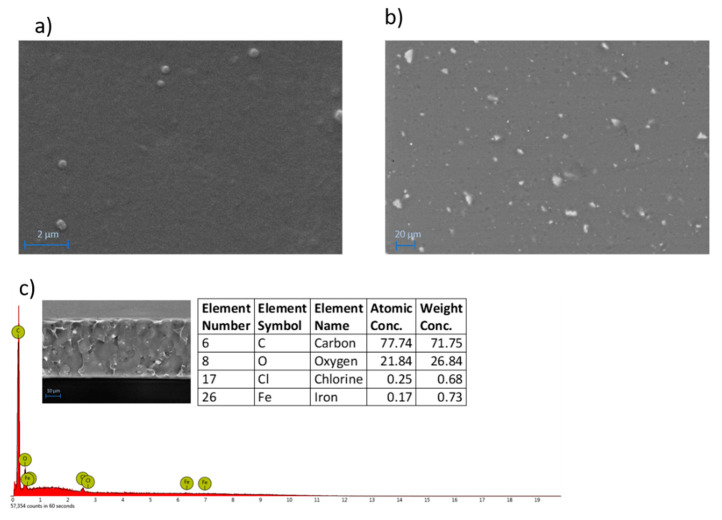
SEM (**a**) and BSD (**b**) images of the surface of M3 membrane (SEM Magnification: 20.00 K×; BSD Magnification: 800×). EDX spectrum with elemental analyses and BSD image cross-section of M3 membrane (**c**).

The surface morphology of the M3 membrane, (Figure 4a) which is representative of all the membranes prepared, presents a dense and compact surface. The addition of Fe-MOF-MW fillers was accompanied by the formation of small particles clearly visible on the surface as white spots (Figure 4b) by BSD analyses. The particles are in the form of small agglomerates ranging from about 1 to 10 μm and uniformly distributed on the membrane surface and along the cross-section (Figure 4c), which presented a dense and compact structure with an overall thickness of 34 ± 3 µm. EDX analyses, carried out along the membrane cross-section, detected the presence of Fe as the characteristic element of the MOF confirming their effective entrapment into the membrane matrix.

**Figure 5:**
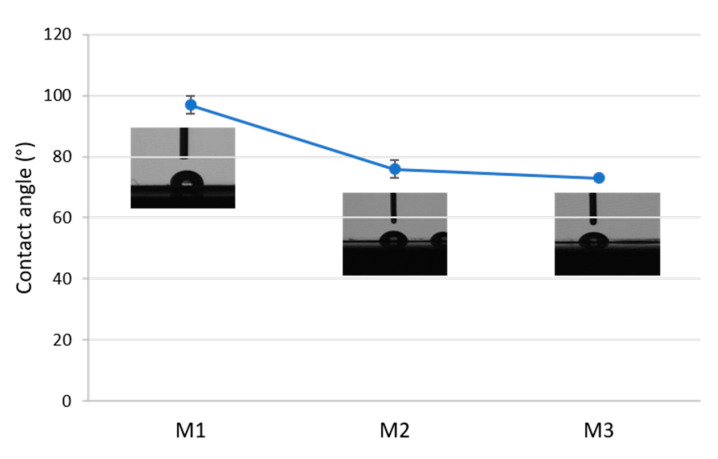
Contact angle of the prepared membranes: M1: pristine PLA. M2, M3: PLA containing Fe-MOF-MW filler.

The results of contact angle measurements on three membranes are presented in Figure 5. The pristine PLA membrane M1 showed a contact angle of about 96°. Membranes containing the Fe-MOF-MW filler (M2 and M3) exhibited a decrease in the contact angle to 72° due to increased hydrophilicity. This effect can be related to the highly hydrophilic nature of MOFs, as reported in the literature [54].

**Figure 6:**
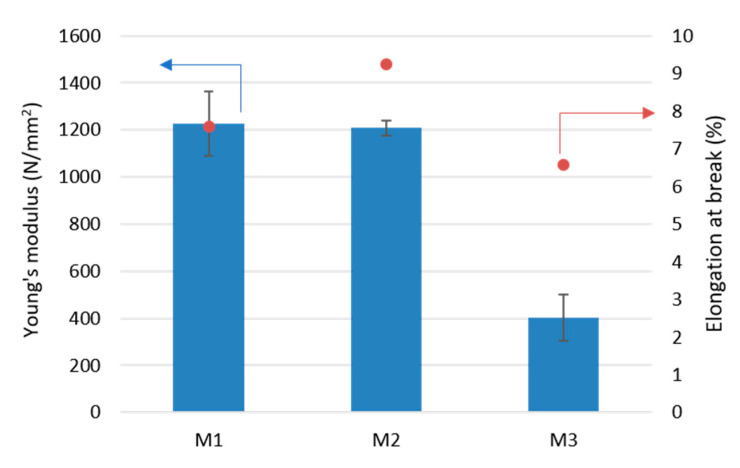
Young’s modulus and elongation at break of investigated membranes.

Figure 6 shows the results of the measurements of two mechanical properties of PLA membranes: Young’s modulus and elongation at break. For the pristine PLA membrane (M1) the results of the Young’s modulus and the elongation at break were 1200 N/mm^2^ and 8%, respectively. Addition of small concentrations of Fe-MOF-MW (0.1 wt%—M2) did not influence significantly the Young’s modulus, which can be explained by the good interfacial adhesion between the PLA polymeric chains and the MOF nanoparticles. The elongation at break of the M2 membrane was slightly increased by about 20% with respect to the M1 pristine membrane. This increase could be attributed to the presence of possible voids which improved the flexibility of PLA/MOF membranes as also observed by others [55,56]. However, a further increase in Fe-MOF-MW concentration (0.5 wt%—M3) caused a drop in Young’s modulus (about 400 N/mm^2^) and elongation at break (6.5%). This effect could be related to a higher agglomeration of MOF, which led to lower reinforcement of the PLA matrix [56].

Due the loss of mechanical properties, as the concentration of Fe-MOF-MW was increased, it was not possible to further increase the concentration of particles into PLA membranes. As the Fe-MOF-MW concentration was, in fact, risen to 0.7 wt%, the membranes were too fragile to be characterised and tested in PV. The addition of Fe-MOF-MW in the range investigated (0.1–0.5 wt%) was adequate to compensate the distortion in the PLA chains responsible of the formation of selective pores keeping, at the same time, acceptable mechanical properties. Higher filler loadings (over 0.5 wt%) are, therefore, responsible of a decrease in membranes mechanical stability, and, probably, of a worsening of the separation properties [57,58].

**Figure 7:**
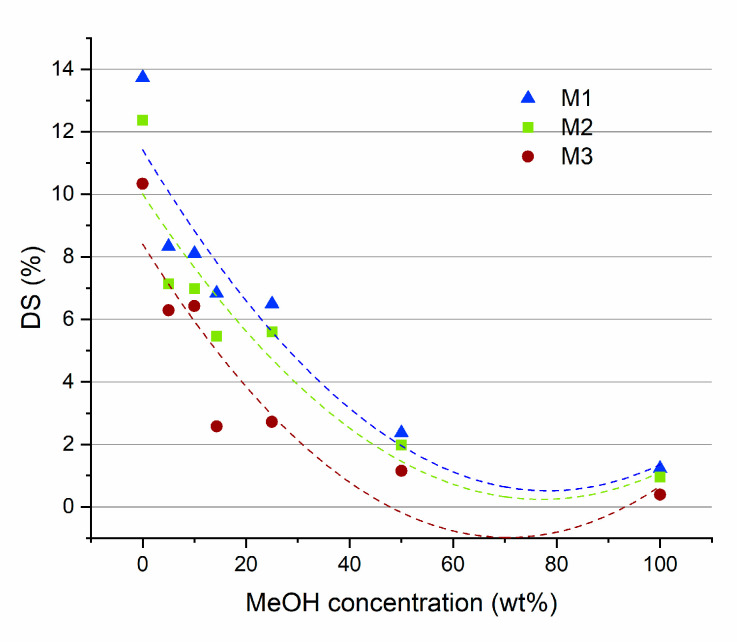
The results of DS measurements for M1, M2 and M3 membranes at different MeOH/MTBE solutions.

Measuring the degrees of swelling (DS) for the M1, M2 and M3 membranes, at different MEOH/MTBE concentrations, yielded the results that are presented in Figure 7. For all these membranes, the highest DS values were observed in pure MTBE (from 10 to 14%). Increasing the concentration of MeOH in the solvents mixture caused a decrease in the measured DS, reaching the lowest values in pure MeOH (about 1.5%). These results can be related to the Hansen solubility parameters of the polymer and the solvents. PLA has a total solubility value (*δt*) of 21.73 which is close to that of MTBE (*δt* = 15.73) with respect to that for MeOH (*δt* = 29.60) [5,59]. Closer solubility values are an indication of a higher affinity between the two species. The higher affinity of PLA for MTBE is reflected in larger swelling of the membrane, in comparison to MeOH.

The presence of Fe-MOF-MW in the PLA membrane induces a decrease in the DS at all MTBE/MeOH ratios. This decrease can be associated with a strong MOF-PLA interaction which may hinder the mobility of polymer chains and thus decrease the free volume of the polymer.

### 3.3. Pervaporation Results

#### 3.3.1. Effect of Temperature

**Figure 8:**
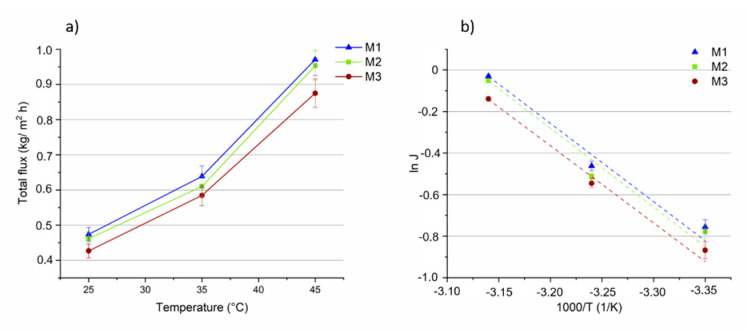
(**a**) The total fluxes through the M1, M2 and M3 membranes as a function of the temperature (vacuum permeate pressure: 0.09 mbar). (**b**) Arrhenius plot of total flux through the membranes.

**Figure 9:**
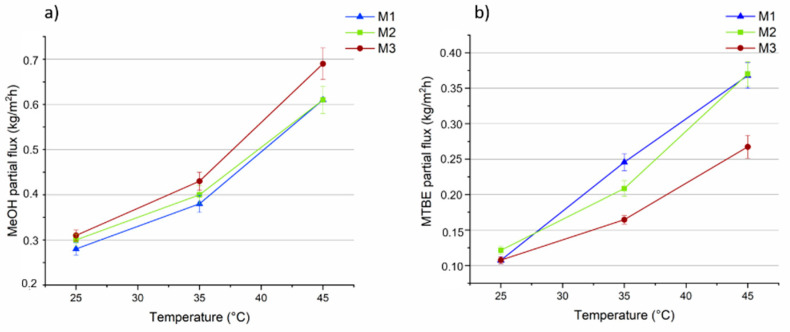
MeOH (**a**) and MTBE (**b**) partial fluxes through the M1, M2 and M3 membranes as a function of the temperature (vacuum permeate pressure: 0.09 mbar).

**Figure 10:**
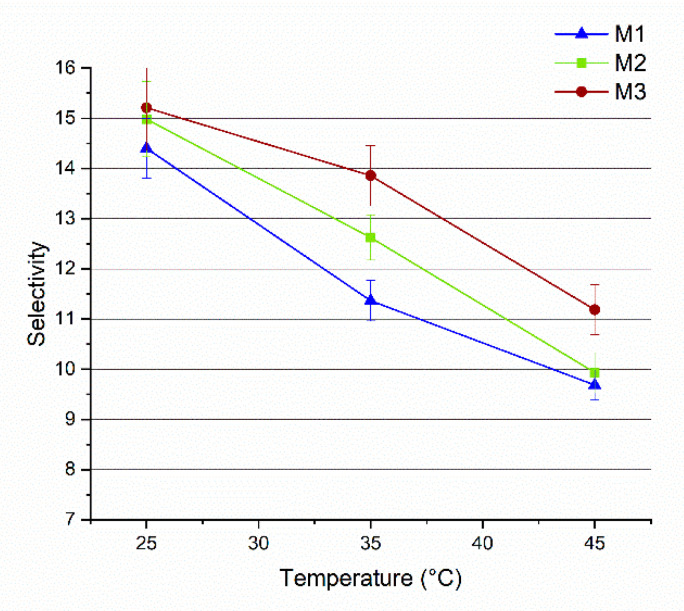
Selectivity of M1, M2 and M3 membranes as a function of the temperature (vacuum pressure: 0.09 mbar).

The influence of temperature (in the range of 25 °C to 45 °C) on the total flux, on the MeOH and MTBE partial fluxes and on selectivity, for the three membranes, is shown in Figure 8, Figure 9 and Figure 10. The feed concentration (at the azeotropic point (14.3 wt% of MeOH)) and the vacuum permeate pressure (0.09 mbar) were kept constant. In Figure 8a, the effect of feed temperature on total flux is reported for all the three membranes.

As expected, the flux through the membranes increased as the feed temperature increased. It can be attributed to a higher thermal motion of the polymer chains producing more free volumes inside the membrane, enhancing the diffusion rate of the permeating molecules and, hence, of the total flux. The total flux slightly decreased (between 1 and 9%) as the concentration of MOF in the membrane was increased. This can be explained by looking at the MeOH and MTBE partial fluxes of the M1–M3 membranes.

Arrhenius plots of total flux as a function of the reciprocal of the absolute temperature shows a linear relationship, (Figure 8b). The apparent activation energies (E_p_), calculated from the slopes of the curves and Equation (5), show nearly similar and positive values (about 28 kJ/mol) for all the three membranes (Table 1), indicating that the permeation flux increases with the temperature, as evidenced by most of the PV studies [60]. The similar results of E_p_ are a consequence of membranes’ similar slopes.

The M3 membrane, loaded with the highest concentration of MOF (0.5 wt%), showed the highest flux for MeOH (up to 0.7 kg/m^2^ h at 45 °C), as shown in Figure 9a, that was about 14% higher in comparison to the unfilled PLA membrane (about 0.6 kg/m^2^ h at 45 °C). However, for the MTBE partial fluxes (Figure 9b) the M1 PLA pristine membrane presented a flux of about 0.37 kg/m^2^ h (at 45 °C) which was 27% higher than that for the M3 membrane (0.27 kg/m^2^ h at 45 °C) contributing to a greater extent to the total flux increase. Arrhenius plots for MeOH and MTBE were drawn based on the data presented in Figure 9a,b, from which the activation energies for both solvents at each kind of membrane were calculated (Table 1).

This data show that at all the investigated temperatures, E_p_ for MeOH is always lower than for MTBE, indicating easier permeation of methanol through the membrane and higher selectivity of the membrane for MeOH. Moreover, the higher E_p_ of MTBE suggests that the permeation of MTBE is more sensitive than MeOH to temperature changes (as observed in other works [61]). In fact, as can be seen from Figure 9, the MTBE flux increased faster than MeOH flux as the feed temperature was increased.

Figure 10 presents a plot of the MeOH selectivity as a function of the temperature. For all three membranes the selectivity decreases at higher temperatures. According to the free volume theory, the temperature increase is accompanied by an increase in the thermal motion of polymer chains in the amorphous regions. This results in larger free volumes which facilitate the diffusion of larger molecules (such as MTBE), thus decreasing the membrane selectivity [57,62]. At all the investigated temperatures, the addition of Fe-MOF-MW had a beneficial effect on membrane selectivity (up to 22% improvement). This may be explained by the creation of preferential pathways for MeOH molecules to get through the membrane. MIL-100 structure has been selected on the basis of its large and permanent porosity, formed by two sets of mesoporous cages (24 Å and 29 Å) accessible by open microporous windows of about 8.6 Å and 4.7–5.5 Å [27]. As demonstrated by the elaboration of N_2_ adsorption data, the Fe-MOF-MW showed the predominance of micropores of 5.5 Å (Figure 2c). Reasonably, MeOH molecules, displaying a kinetic molecular diameter of about 0.4 Å, are more likely to pass through both types of apertures in contrast to MTBE molecules which have a bigger kinetic molecular diameter of about 6.2 nm [3] and that can be excluded by molecular sieves. Such structures were reported in the literature to be able to store and release more than 40% of MeOH [63]. In fact, despite the amphiphilic nature of the MOFs, composed of polar (metallic clusters, carboxylate groups) and a polar (aromatic linker) fractions, this structure demonstrated an overall highly hydrophilic character [54] which contribute to improving the hydrophilicity of the overall membrane, as confirmed by contact angle results. The polarity indexes of MeOH and MTBE are 0.762 and 0.124, respectively [40,64]. For this reason, the higher polarity makes the MeOH molecules more favoured to permeate through the hydrophilic Fe-MOF-MW loaded membranes with respect to MTBE.

Unfortunately, it was not possible to further improve the concentration of Fe-MOF-MW into PLA membranes due to the loss of membranes’ mechanical properties which made them unsuitable for PV testing.

#### 3.3.2. Effect of Permeate Vacuum Pressure

**Figure 11:**
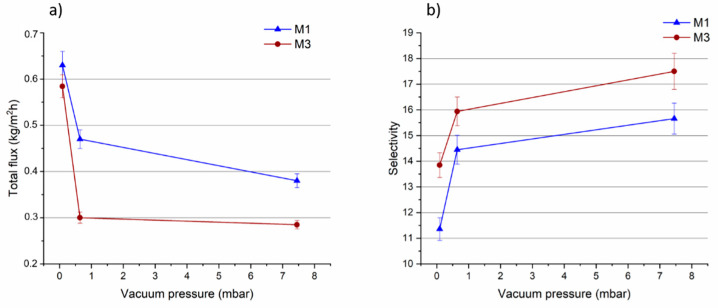
(**a**)Total flux and selectivity (**b**) of M1 and M3 membranes as a function of the vacuum pressure (temperature: 35 °C).

The effect of permeate pressure on the performance of PLA pristine membrane and on the M3 membrane, which exhibited the best selectivity was also evaluated. The measurement was done at constant temperature of 35 °C while the vacuum at the permeate side was varied (0.09, 0.64 and 7.45 mbar). Figure 11a shows that the total flux through the M1 and M3 membranes decreased as the vacuum permeate pressure was decreased (from 0.09 to 7.45 mbar). Since the driving force for permeation is the difference in vapour pressure at the two sides of the membrane, decreasing the vacuum leads to a decline of membrane total flux [2,65].

The selectivity, on the contrary, increased for both membranes as the vacuum pressure was decreased (as shown in Figure 11b).

The difference in the driving force, caused by changes of the vacuum, can affect the permeating molecules. MTBE molecules were more negatively influenced by the lower vacuum degree in comparison to MeOH molecules, leading to an enhancement in membrane selectivity. The results presented in Table 2, show that the decrease in MTBE partial flux, as a function of the vacuum pressure, was more pronounced than MeOH partial flux, for both membranes. Such trend has been recently reported also for PLA membranes [5].

The M3 membrane loaded with 0.5 wt% of MOF showed the best selectivity at all the investigated pressures in comparison to the PLA pristine membrane.

To the best of our knowledge, this is the first time that PLA based MMMs have been developed and employed for PV. For this reason, it is difficult to make a direct comparison between the performance obtained in this work and literature data. Other materials have been explored so far and reported in the literature for such organic/organic separation. A comparison of PLA+ Fe-MOF-MW performance with other studies reported in literature is reported in Table 3.

Wang et al. [66], for instance, employed graphene oxide (GO) and poly(methylene bisacrylamide aminoethyl piperazine) (HPMA) on a ceramic substrate for MeOH/MTBE separation reaching a selectivity up to 4500. Cellulose acetate (CA) and polyvinyl pyrrolidone (PVP)-based membranes have been also reported by Wu et al. [67] for the same type of separation reaching a selectivity of 420.

However, the results of this work are comparable or superior to the performance exhibited by other membrane materials such as Matrimid^®^ [2], poly(ether ketone) (PEEK-WC) [3] and chitosan [68]. The results here presented show that the addition of specific MOFs (even at very low concentrations) can effectively improve the performance of PLA membranes in terms of selectivity, without compromising on the total flux. More importantly, a biopolymer has been used to achieve such kind of separation in combination with MOFs produced according to a solvent free protocol. The outcomes stemming from this work can be pivotal in opening up new horizons to the production of a new class of highly performing membranes based on the use of biomaterials.

Certainly, there is still room for improvement and much more studies are needed for further improving PLA membrane performance in order to make them competitive with respect to more efficient materials. In this frame, the design and synthesis of new MOFs exhibiting extreme selectivity for target compounds can open up new horizons to the production of a new class of highly performing membrane materials.

## 4. Conclusions

In this study, novel MMMs have been produced by solvent evaporation. These membranes were tested for the separation of a MeOH/MTBE azeotropic mixture via PV. In order to make the membrane preparation procedure as sustainable as possible, a biopolymer (PLA) and an iron-MOF, produced according to a much more environmentally safe and solvent-free protocol. The MOF was obtained in water within 20 min using microwave’ energy, yielding spherical particles with a narrow distribution. These particles had permanent porosity with a total pore volume of 0.96 cm^3^/g and a relatively narrow micropore size distribution centred on 5.5 A. The incorporation of Fe-MOF-MW into PLA matrix was demonstrated with SEM and BSD analyses and showed the MOF nanoparticles as small agglomerates, uniformly distributed on the membrane surface and along the cross-section. The presence of MOF as filler has important effects on membrane wettability, swelling degree and mechanical properties. When the MMMs were tested for PV, the Fe-MOF-MW fillers favoured the permeation of MeOH molecules through the membrane matrix leading to an improvement in the membrane selectivity. Arrhenius relationship showed that the flux has a linear dependency on the applied temperature, and the E_p_ calculated values confirmed the easier permeation of MeOH molecules through the membrane. At the concentration of 0.5 wt% of Fe-MOF-MW, the best performance in terms of selectivity was obtained with an improvement of 22% respect to the pristine unfilled PLA membrane. The particular architecture and window dimensions of selected MOF were supposed to create preferential pathways for the permeation of the smaller MeOH molecules fostering the separation of the investigated organic/organic mixture.

## Figures and Tables

**Table 1:** Apparent activation energies for total flux, MeOH and MTBE partial. Fluxes of M1, M2 and M3 membranes.

Membrane	Activation Energies (E_p_) Values (kJ/mol)		
	Total	MeOH	MTBE
**M1**	28.48	30.70	50.66
**M2**	28.49	27.90	44.30
**M3**	28.65	30.60	37.63

**Table 2:** Total flux, MeOH and MTBE partial fluxes and selectivity for M1 and M3 membrane at different vacuum pressures (temperature: 35 °C).

	Pressure (mbar)	Total Flux (kg/m^2^ h)	MeOH Partial Flux (kg/m^2^ h)	MTBE Partial Flux (kg/m^2^ h)	Selectivity
**M1**	0.09	0.63	0.38	0.24	11.3
	0.64	0.47	0.22	0.14	14.4
	7.45	0.38	0.28	0.10	15.6
**M3**	0.09	0.58	0.44	0.16	13.8
	0.64	0.3	0.32	0.08	15.9
	7.45	0.28	0.21	0.07	17.5

**Table 3:** Comparison of the PLA + Fe-MOF-MW performance with other studies in literature.

Membrane Material	Concentration of MeOH/MTBE Mixture	Operative Conditions	Total Flux (kg/m^2^ h)	Selectivity	Reference
**HMPA + GO (0–20 wt%)**	10 wt% MeOH 90 wt% MTBE	T = 40 °C Vacuum pressure = 3 mbar	0.2–0.4	2400–4500	[66]
**CA (85 wt%) + PVP (15 wt%)**	20 wt% MeOH 80 wt% MTBE	T = 40 °C Vacuum pressure = 3 mbar	0.43	411	[67]
**Matrimid^®^ 5218**	14.3 wt% MeOH 85.7 wt% MTBE	T = 35 °C Vacuum pressure = 0.054 mbar	0.06	17.7	[2]
**PEEK-WC**	14.3 wt% MeOH 85.7 wt% MTBE	T = 30 °C Vacuum pressure = 6 mbar	0.02	14	[3]
**Chitosan**	17.5 wt% MeOH 82.5 wt% MTBE	T = 25 °C Vacuum pressure = 4–6 mbar	0.4	14	[68]
**Polyamide + Al_2_O_3_ (10 wt%)**	50 wt% MeOH 50 wt% MTBE	T = 30 °C Vacuum pressure = 4–6 mbar	15 *	20	[69]
**CA + HZSM5 (0–1 wt%)**	20 wt% MeOH 80 wt% MTBE	T = 30 °C Vacuum pressure = 3.3 mbar	0.16–0.3	120–350	[70]
**CA + ZnO (0–14 wt%)**	31 wt% MeOH 69 wt% MTBE	T = 40 °C Vacuum pressure below 5 mbar	1–4.5 *	200–800	[71]
**PLA+ Fe-MOF-MW (0.5 wt%)**	14.3 wt% MeOH 85.7 wt% MTBE	T = 35 °C Vacuum pressure = 7.45 mbar	0.28	17.5	This work

* Flux normalized by thickness.

## Data Availability

Data is contained within the article.
